# Comparing the Success Rate of Dacryocystorhinostomy With and Without Silicone Intubation: A Trial Sequential Analysis of Randomized Control Trials

**DOI:** 10.1038/s41598-017-02070-y

**Published:** 2017-05-16

**Authors:** ChuanQi Xie, Lingling Zhang, Yang Liu, Hong Ma, Shuzhen Li

**Affiliations:** 1 Department of Ophthalmology, First People’s Hospital of Shangqiu, Henan, China; 2 Shangqiu Medical College, Shangqiu, Hanan China

## Abstract

A previous meta-analysis reported no benefit for silicone intubation during dacryocystorhinostomy. However, the power of this meta-analysis was 0.274. Therefore, the benefit of silicone intubation remains controversial. We undertook a cumulative meta-analysis to evaluate the success rate of dacryocystorhinostomy (DCR) with and without the use of a stent. Pubmed, EMBASE and the Cochrane Library were searched. Statistical power and trial sequential analyses were performed according to the result of the meta-analysis. Twelve randomized controlled trials involving 969 cases met the inclusion criteria. The success rates of DCR with and without intubation were significantly different (p = 0.006). The success rates of external DCR (EX-DCR) with and without intubation were also significantly different based on subgroup analysis (p = 0.002). The cumulative z-curves crossed the O’Brian-Fleming boundaries. There were no significant differences in the success rate in the endonasal endoscopic DCR (EN-DCR) subgroup or the occurrence of postoperative complications between the two groups based on the meta-analysis, and the z-curve did not intersect any trial sequential analysis boundaries. DCR with intubation achieved better results than DCR without intubation, especially in the EX-DCR subgroup. Differences in the success rate in the EN-DCR subgroup and postoperative complications between the two groups were underpowered to reach a conclusion.

## Introduction

Dacryocystorhinostomy (DCR) is the most popular operation for treating nasolacrimal duct obstruction or chronic dacryostenosis^1^. DCR is a surgical procedure to create drainage between the lacrimal sac and the nasal cavity^2^. DCR procedures include standard external DCR (EX-DCR), non-laser endonasal endoscopic DCR (EN-DCR), and endonasal endoscopic laser DCR (LA-DCR). Beginning in the 1970s, ophthalmologists began to favour DCR with silicone intubation over DCR without intubation^3^. They advocated its use and reported an increased postoperative patency rate because of maintenance of the opening of the ostium^4^. However, other studies reported a higher failure rate when using a silicone stent because of granulomatous inflammation^5^. The role of silicone intubation during DCR surgery has been discussed several times in the recent literature, with conflicting opinions^6, 7^. The aim of this study was to evaluate the success rate with and without the use of a stent during DCR and to compare the results with those of previously published studies. We hope the results of this study will give clinicians a more definitive set of guidelines and indications for tube use.

## Materials and Methods

**Figure 1:**
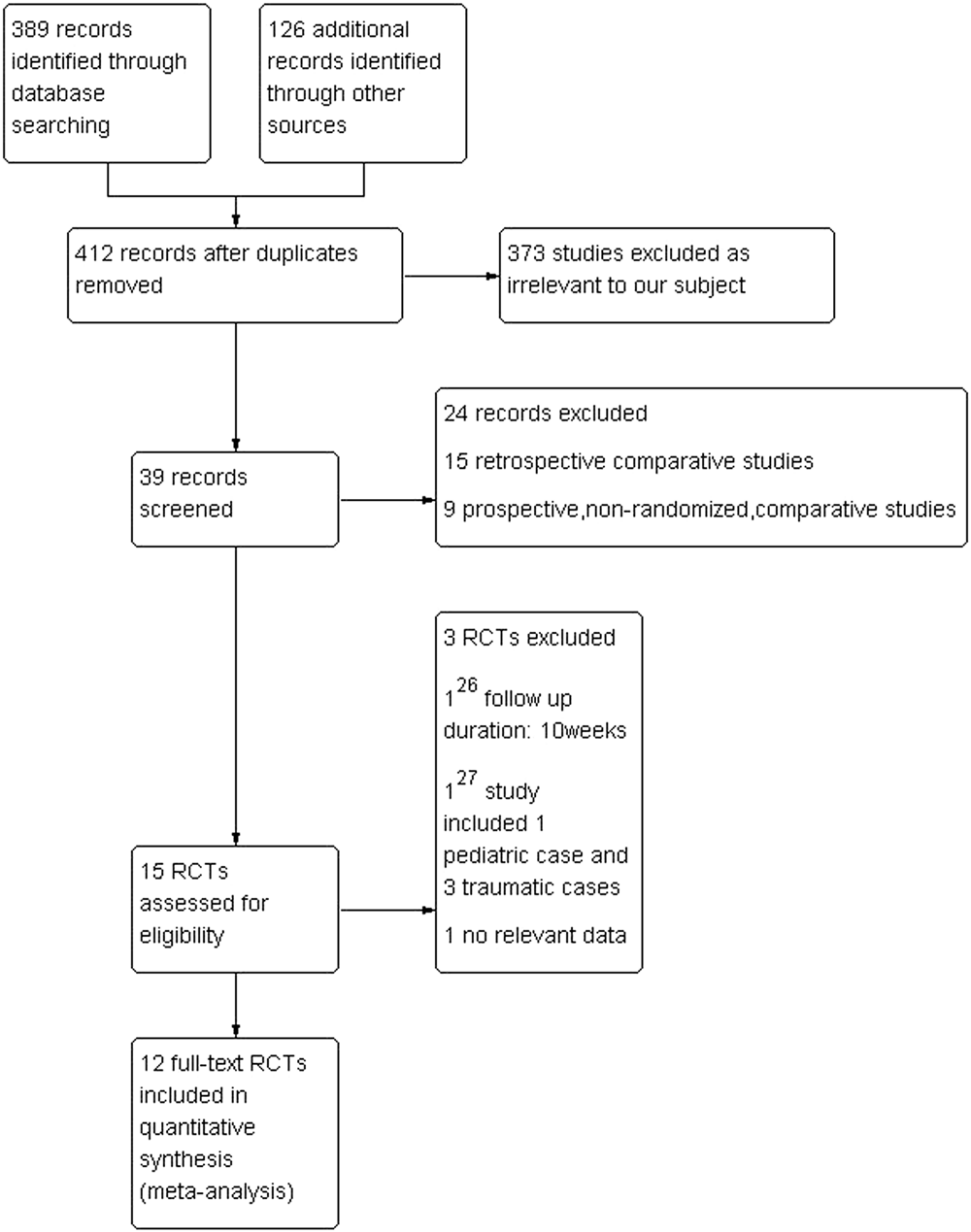
Study flow diagram^26, 27^.

The following electronic databases were searched from January 1, 1990 to June 1, 2016: PubMed, EMBASE, the Cochrane Controlled Trials Register, Ovid, ScienceDirect, NGC, and EBSCO. We developed a search strategy including the following terms: “dacryocystorhinostomy”, “silicone intubation”, “stent”, “nasolacrimal duct obstruction” and “dacryocystitis”. The literature search only included English-language articles. The titles of all articles were read, and the relevant abstracts were evaluated. The full articles were retrieved if the title, abstract, or both of a study seemed to meet the objective of this review. The reference lists of original reports and review articles retrieved through the search were reviewed for additional studies not yet included in the computerized databases (Fig. 1).

Study inclusion criteria: 1. Design: Only randomized controlled trials were included. 2. Population: Adult patients who were confirmed to have nasolacrimal duct obstruction or chronic dacryocystitis based on the symptoms of epiphora and the results of lacrimal irrigation. 3. Intervention: DCR with silicone intubation versus DCR without silicone intubation were compared. DCR techniques could include EX-DCR, LA-DCR or EN-DCR. 4. Follow-up duration: At least 6 months of follow-up was required. 5. Outcome measures: The success rates of each group based on subjective or objective assessments were included as outcomes. Patients who had lacrimal sac tumours, canalicular obstruction, a history of lacrimal surgery and traumatic injury to the ocular or nasal regions were excluded. Studies were further excluded if the study cohorts included paediatric cases.

Data extraction was performed according to a customized protocol. The following categories of information were extracted: study characteristics (author, year and country of publication), patient characteristics (mean age, gender, number of participants and withdrawals) and intervention characteristics (type of surgical intervention, follow up duration, extubation time, outcomes and complications).

The risk of bias in each included study was assessed according to the Cochrane Collaboration tool for assessing the risk of bias^8^, which is structured into seven domains: random sequence generation, allocation concealment, blinding of participants and personnel, blinding of outcome assessment, incomplete outcome data, selective reporting and other sources of bias.

Document screening, information extraction and qualitative assessment were performed by two reviewers independently. Any disagreement was resolved by discussion or consensus involving a third reviewer when necessary.

### Statistical Analysis

The statistical analysis was performed using the RevMan software package (version 5.3, The Cochrane Collaboration, London, England). A pooled risk ratio (RR) was calculated with a 95% confidence interval (CI). Statistical heterogeneity was assessed via *I* ^*2*^ statistics. The fixed-effects models were accepted if *I* ^*2*^ < 50%. Otherwise, random-effects models were used^9^. Subgroup analysis was performed based on the type of DCR technique. A *p-*value < 0.05 was considered statistically significant.

Cumulative meta-analysis models were performed using the Stata software package (version 11.0, Stata Corporation, College Station, TX, USA). Publication bias was assessed via Egger *et al*. regression asymmetry tests^10^ and Begg and Mazumdar’s adjusted rank correlation tests^11^. A funnel plot was also constructed to display publication bias. The statistical power was calculated using the Power and Precision software package (version 4, Biostat, Inc., Chicago, Illinois, USA), when negative results were obtained^12^.

The trial sequential analyses were performed using the TSA software package (version 0.9 beta, The Copenhagen Trial Unit, Copenhagen, Denmark), based on estimated information size with a risk of type I error of a = 0.05 and a risk of type II error of β = 20%^13^.

## Results

**Figure 2:**
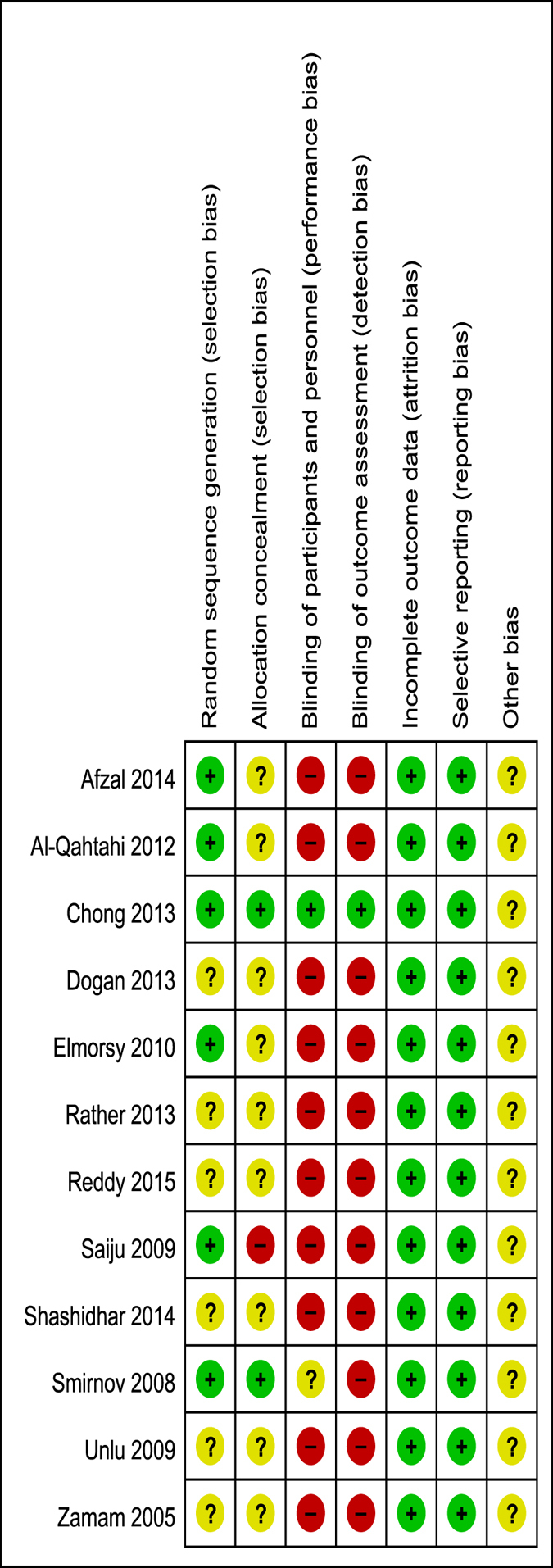
Risk of bias summary: the review authors’ judgements about each risk of bias item for each included study.

A total of 412 records were identified, and 12 RCTs^14–25^ were included in the quantitative analysis. The studies included a total of 969 cases. Of these, 494 cases had undergone DCR with silicone intubation, and 475 cases had undergone DCR without silicone intubation. (Table 1). Each included study was assessed using the Cochrane Collaboration’s tool for assessing the risk of bias (Fig. 2).

**Table 1:** Characteristics of the Included Studies.

Author	Year	County	Study design	Surgical technique	Mean age	Mean follow-up	No. of patients(eyes)	Gender (M/F)	Withdrawal (eyes)	Silicone removed	Outcomes measured
Zaman	2005	Pakistan	RCT	EX-DCR	31–60	12 months	80(80)	30/50	0	6 months	success rate, complication
Smirnov	2008	America	RCT	EN-DCR	64	6 months	42(46)	9/37	0	2 months	success rate, complication
Unlu	2009	Turkey	RCT	EN-DCR	55.4(32–73)	99.6 months	42(44)	9/29	6	2 months	success rate, complication
Saiju	2009	America	RCT	EX-DCR	41(18–82)	6 months	100(100)	22/78	48	6 months	success rate
Elmorsy	2010	Egypt	RCT	EX-DCR	45.6	9 months	46(46)	29/17	0	3 months	success rate, complication
Al-Qahtahi	2012	Saudi Arabia	RCT	EN-DCR	51.8(18–72)	12 months	173(173)	67/106	0	4 mouths	success rate
Dogan	2013	Turkey	RCT	LA-DCR	62(39–77)	18.1 months	80(88)	13/67	6	6 months	success rate, complication
Chong	2013	China	RCT	EN-DCR	64(39–92)	12 months	118(128)	16/102	2	2 months	success rate, complication
Rather	2013	India	RCT	EX-DCR	NA	12 months	200(200)	NA	0	1 months	success rate, complication
Afzal	2014	Pakistan	RCT	EX-DCR	42.49(20–65)	6 months	80(80)	16/64	0	NA	success rate
Shashidhar	2014	India	RCT	EN-DCR	17–75	6 months	57(62)	15/42	0	1.5 months	success rate, complication
Reddy	2015	India	RCT	EN-DCR	NA	6 months	20(20)	NA	0	1.5 months	success rate, complication

**Figure 3:**
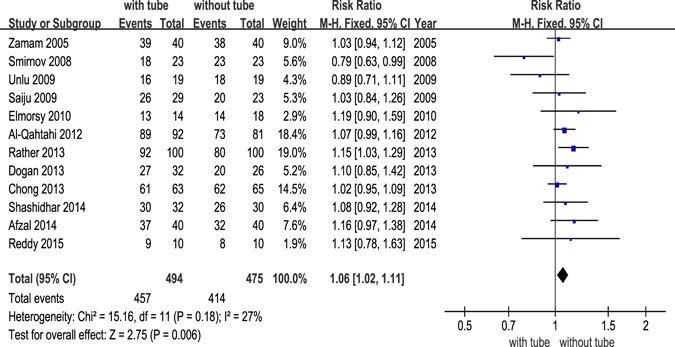
Forest plot: comparison of success rate between DCR with and without silicone intubation.

All studies provided data regarding the success rate of both groups. The heterogeneity test indicated no significant heterogeneity (*I*_2_ = 27%, *p* = 0.18), and a fixed-effects model was adopted. The forest plot revealed that DCR with silicone intubation had a much higher rate of success than DCR without silicone intubation. The difference was statistically significant (RR, 1.06; 95% CI [1.02–1.11], *p* = 0.006). The statistical power was 0.788 (Fig. 3).

**Figure 4:**
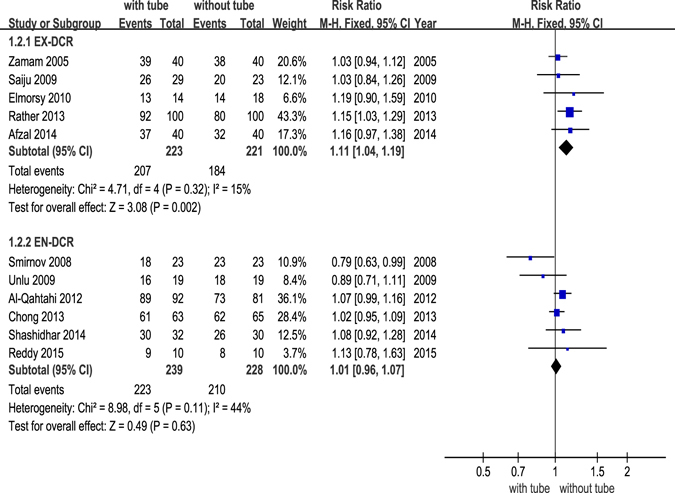
Forest plot: subgroup analysis of the success rate between DCR with and without silicone intubation.

We divided the studies into 3 groups depending on surgery type (EX-DCR, EN-DCR or LA-DCR) to perform subgroup analysis. The forest plot revealed that there was a significant difference in the EX-DCR group (*p *= 0.002) but no significant difference in the EN-DCR group (*p* = 0.63). The powers of the EX-DCR and EN-DCR subgroup analyses were 87.2% and 7.9%, respectively (Fig. 4). Only one study included in the LA-DCR group and reported that there was no significant difference between the success rates of the two groups (*p* = 0.769).

**Figure 5:**
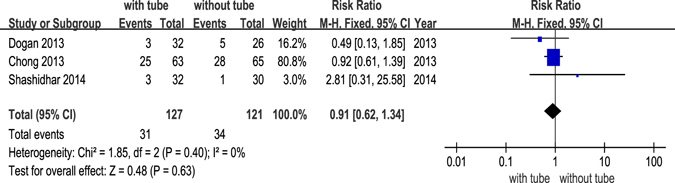
Postoperative complication (granulation) of dacryocystorhinostomy with or without silicone intubation.

**Figure 6:**
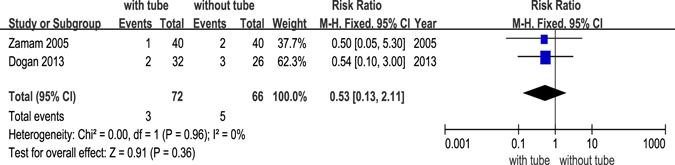
Postoperative complication (infection) of dacryocystorhinostomy with or without silicone intubation.

**Figure 7:**
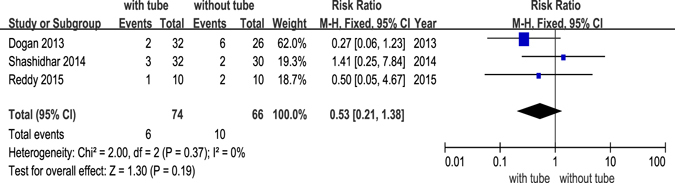
Postoperative complication (adhesion) of dacryocystorhinostomy with or without silicone intubation.

Of 12 RCTs, 9 studies^14–16, 18, 20–22, 24, 25^ reported postoperative complications, including granulation tissue formation, adhesion, infection, haemorrhage and other complications that were considered to be related to the silicone tube, such as punctual/canalicular laceration, tube displacement or loss and conjunctival irritation. Granulation tissue formation after DCR was only reported in 3 studies^20, 22, 24^, infection was reported in 2 studies^14, 20^, and adhesion was reported in 3 studies^20, 24, 25^. The forest plots all revealed no significant differences between DCR with and without silicone intubation (Figs 5,6 and 7). The powers of the three studies were 9.3%, 13.5% and 18.1%, respectively.

**Figure 8:**
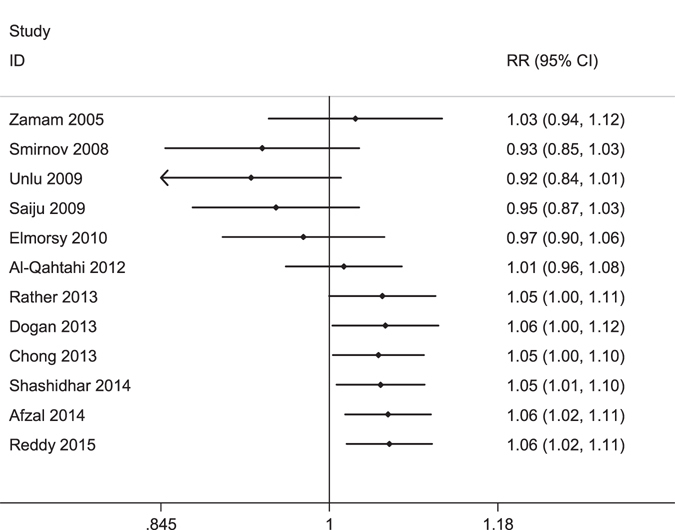
Forest plot of the cumulative meta-analysis.

Sequential cumulative meta-analysed results for each year were calculated from 2005, and the overall effect of success rate began to have statistical significance in 2013. Figure 8 shows the results of the updated cumulative meta-analysis in chronological order.

**Figure 9:**
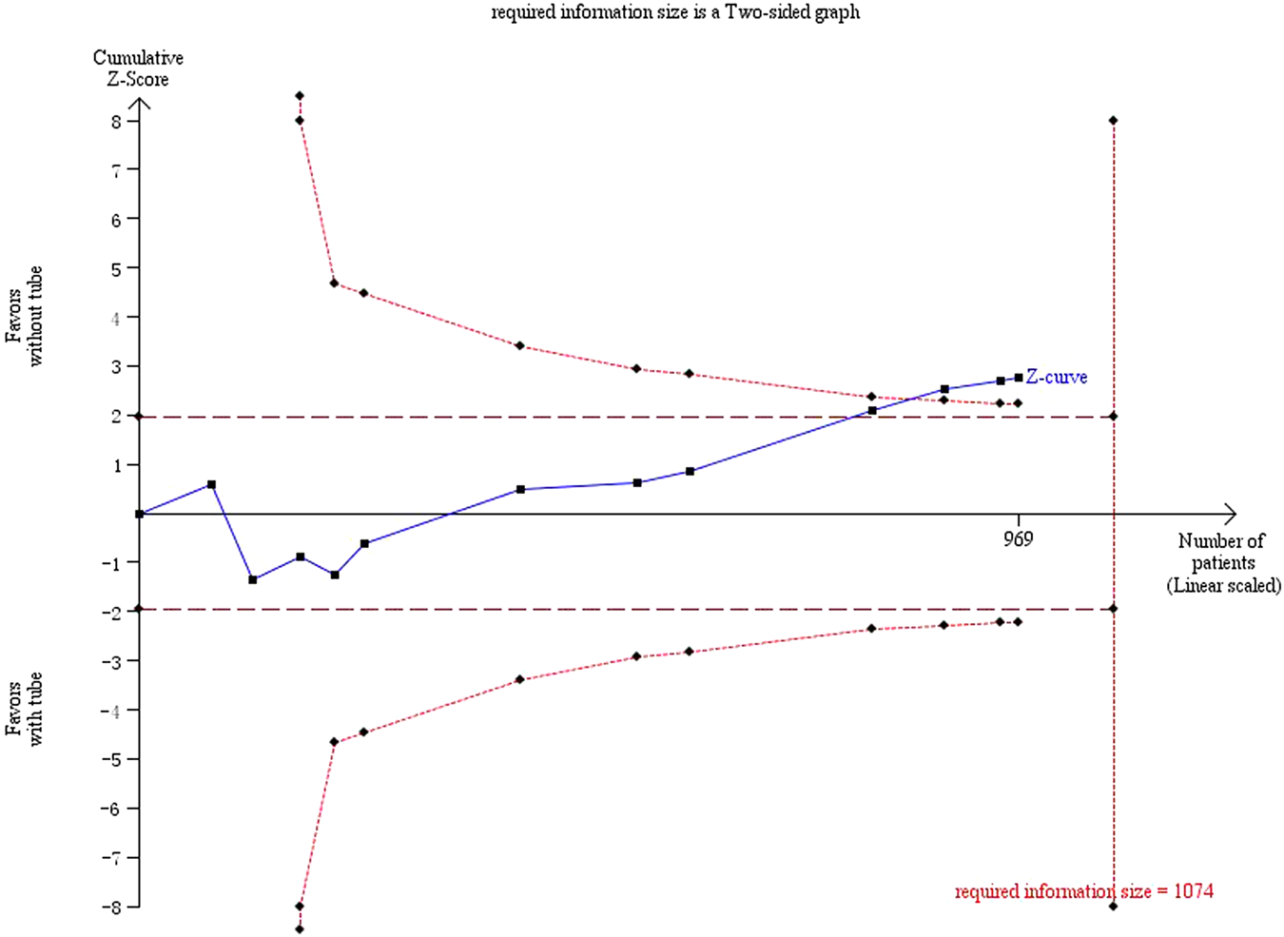
Trial sequential analysis of 12 included studies.

**Figure 10:**
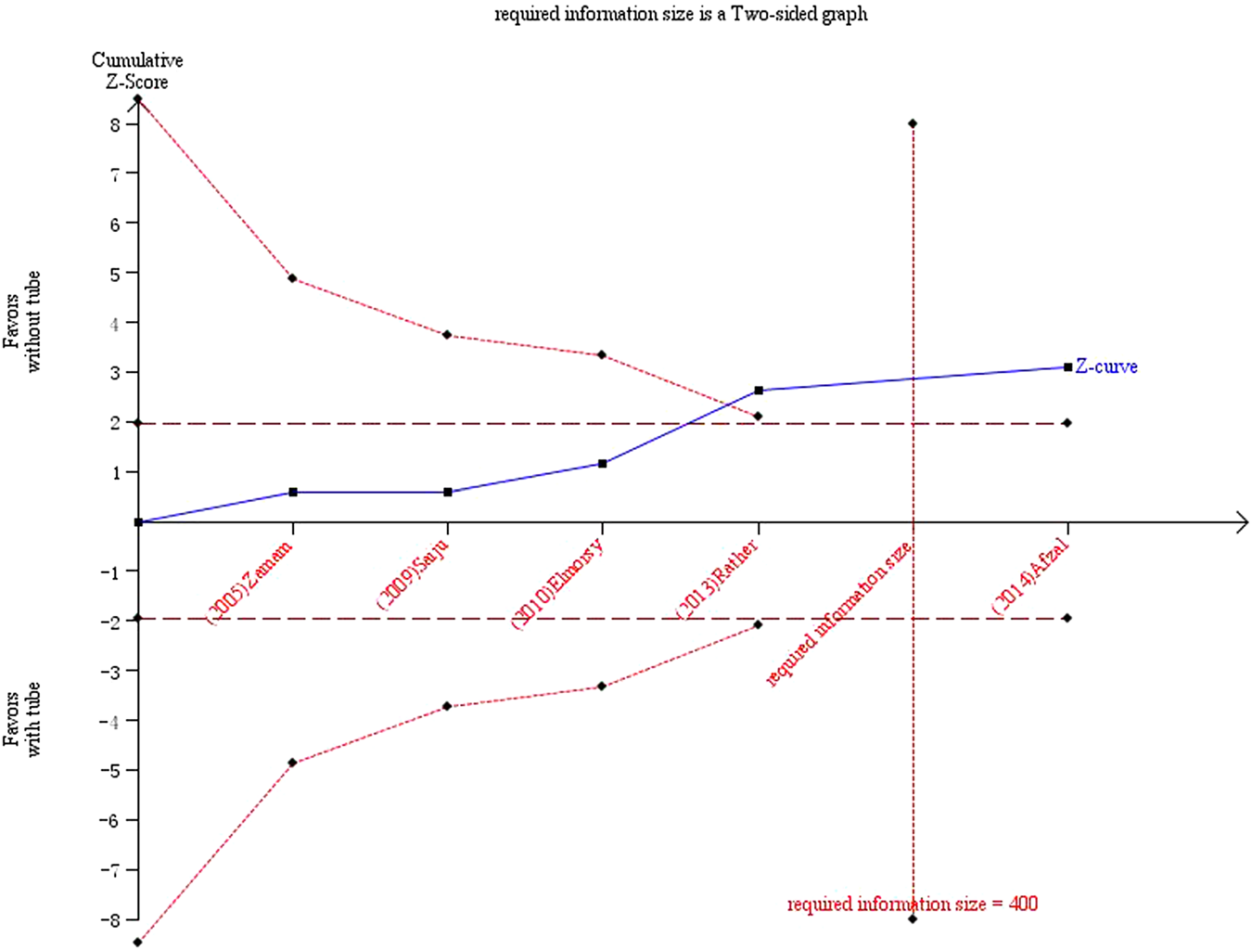
Trial sequential analysis of the EX-DCR subgroup.

**Figure 11:**
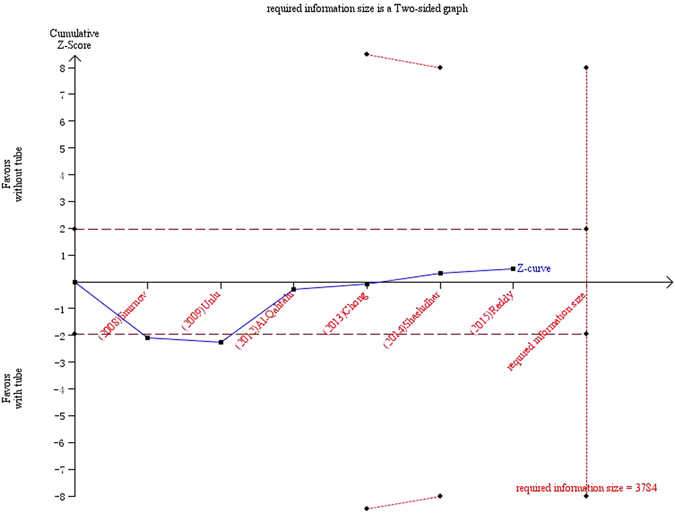
Trial sequential analysis of the EN-DCR subgroup.

In Figs 9 and 10, the cumulative z-score reached significance by crossing both the conventional boundaries and the O’Brian-Fleming boundaries, thus demonstrating the significant benefit of silicone intubation during EX-DCR. In the EN-DCR subgroup, the z-curve did not intersect any TSA boundaries, which indicates that the meta-analysis is underpowered to reach a conclusion (Fig. 11).

**Figure 12:**
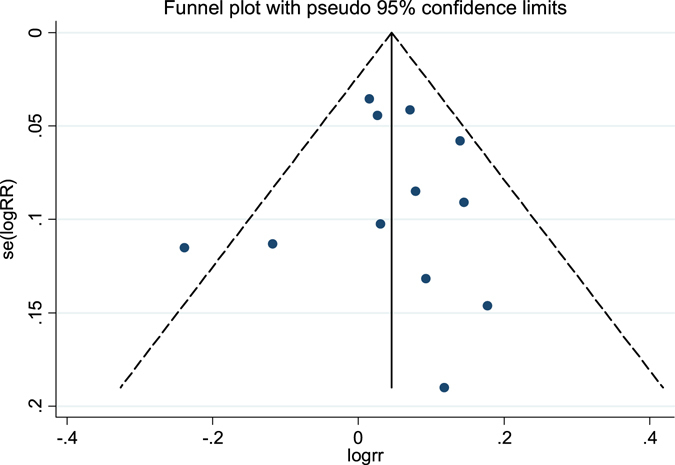
Funnel plot of publication bias.

The publication bias of the study is revealed by the funnel plot (Fig. 12). There was no evidence of publication bias (Begg’s test, *p* = 0.891; Egger’s test, *p* = 0.988).

## Discussion

In 2011, a previous meta-analysis^6^ regarding DCR with and without a silicone tube for the treatment of nasolacrimal duct obstruction reported equal success rates between DCR with and without intubation. The meta-analysis indicated that no benefit was found for silicone stent intubation in primary DCR. However, a growing number of prospective comparative studies published from 2010 onward have reported that the use of silicone intubation in primary DCR increased the success rate of DCR without intubation, although some increases had no statistical significance^28, 29^. Rather and Singh^21^ also conducted a large, randomized controlled trial and demonstrated that silicone intubation in DCR prevented the closure of the ostium, thereby enhancing the success rate of DCR. The previous meta-analysis only included 4 RCTs, and for the given effect size (population proportions 0.892 versus 0.943), sample size (111 and 105) and alpha (0.05, 2-tailed), the power of the meta-analysis was 0.274. The role of silicone intubation during DCR surgery was still undetermined. Therefore, we performed a cumulative meta-analysis to evaluate the success rate with and without the use of a stent in DCR and to compare the results with those of previously published studies.

This cumulative meta-analysis suggested that compared with DCR without intubation, DCR with intubation had a much better rate of success after surgery, especially in the EX-DCR subgroup. The difference was statistically significant [RR, 1.06; 95%CI (1.02–1.11), *p* = 0.006]. The conclusion was completely opposite that of the previous meta-analysis. The previous meta-analysis included fewer studies and had low statistical power, which may explain the different results. When a negative result is obtained, it is important to consider the power of the study^30^. Otherwise, investigators can make a type II error, and treatments that may be of benefit may be discarded.

Meta-analysis is a statistical methodology that combines the results of several independent studies considered by the analyst to be ‘combinable’. It acts to increase the sample size, reduce the random error and enhance the statistical power when the studies included are underpowered^31^. Of the 12 RCTs included in this cumulative meta-analysis, the results of 10 of them were negative. In this article, the results of the cumulative meta-analysis and the EX-DCR subgroup analysis were positive, and the statistical powers of the two analyses were 78.4% and 87.2%, respectively. Because cumulative meta-analyses are prone to produce spurious *p* < 0.05 because of the repeated testing of significance as trial data accumulate, trial sequential analysis should establish when there is firm evidence in a cumulative meta-analysis. The cumulative z-score crossed the O’Brian-Fleming boundaries, and the accrued information size in the EX-DCR subgroup was more than the required information size, which suggested preliminary termination of a clinical trial of the same type. Therefore, this study provided more convincing evidence of the significant benefits of silicone intubation during EX-DCR. In the EN-DCR subgroup, the statistical power of the subgroup analysis was 7.9%, and the z-curve did not intersect any TSA boundaries, which indicates that the meta-analysis was underpowered to reach a conclusion. To definitively determine whether silicone intubation during EN-DCR is beneficial, a large sample or multicentre, randomized, prospective intervention trial is required. The sample size based on trial sequential analysis should be 3784 patients (1892 in each group).

The common complications after surgery were intranasal tissue granulation, adhesion, infection, haemorrhage and other complications that were considered to be related with silicone tube, including punctual/canalicular laceration, tube displacement or loss and conjunctival irritation^32^. The opinion that the silicone tube itself may stimulate tissue granulation was controversial. Unlu *et al*.^33^ suggested that silicone intubation as a foreign inorganic material may predispose the patient to granulation formation with subsequent rhinostomy closure. The ostial size reduction has been reported by *Longari et al*. in higher percentage in the stent group, mainly due to peristomal granuloma, scar tissue formation, and turbinoseptal synaechia^34^. Owing to data restrictions regarding postoperative complications, which many included trials did not provide, this study only analysed complications quantitatively in term of tissue granulation hyperplasia, infection and adhesion. Postoperative complications such as canalicular laceration and tube displacement or loss could not be incorporated into the meta-analysis. The results of the meta-analysis indicated that the use of a silicone tube did not increase the risk of tissue granulation, infection or adhesion. However, the statistical powers for each complication were, respectively, 9.3%, 13.5% and 18.1%. Therefore, the conclusion that the silicone tube itself may stimulate tissue granulation is still unconfirmed.

In conclusion, this cumulative meta-analysis revealed that the success rate of DCR with silicone tubing was significantly better than that of DCR without silicone tubing, especially in the EX-DCR subgroup. The results indicated that silicone intubation was beneficial in treating nasolacrimal duct obstruction during external DCR. Although the meta-analyses revealed no significant differences in terms of success rates or postoperative complications, such as tissue granulation, infection and adhesion, in the EN-DCR subgroup, the meta-analysis was underpowered to reach a conclusion based on statistical power analysis and trail sequential analysis.

### Limitations of this study

This meta-analysis only included twelve RCTs, we did not obtain unpublished study data, and the sample size was small. The type of DCR, the timing of tube removal, the follow-up time and the measurement indicators were not totally consistent across the RCTs. Only a few RCTs had recorded details of postoperative complications. These factors may cause bias. Because of the existing restrictions, it is suggested that a multicentre, large-sample, randomized controlled clinical study be performed to provide more convincing evidence of the efficacy of silicone intubation in EN-DCR for PNLDO.
