# Author Correction: Comparing the Success Rate of Dacryocystorhinostomy With and Without Silicone Intubation: A Trial Sequential Analysis of Randomized Control Trials

**DOI:** 10.1038/s41598-018-37134-0

**Published:** 2018-12-12

**Authors:** ChuanQi Xie, Lingling Zhang, Yang Liu, Hong Ma, Shuzhen Li

**Affiliations:** 1 Department of Ophthalmology, First People’s Hospital of Shangqiu, Henan, China; 2 Shangqiu Medical College, Shangqiu, Hanan, China

Correction to: *Scientific Reports* 10.1038/s41598-017-02070-y, published online 16 May 2017

This Article contains an error in the labelling of the forest plots in Figures 3 and 4 where ‘with tube’ and ‘without tube’ are inverted. The correct Figures 3 and 4 appear below as Figures 1 and 2 respectively.

**Figure 1:**
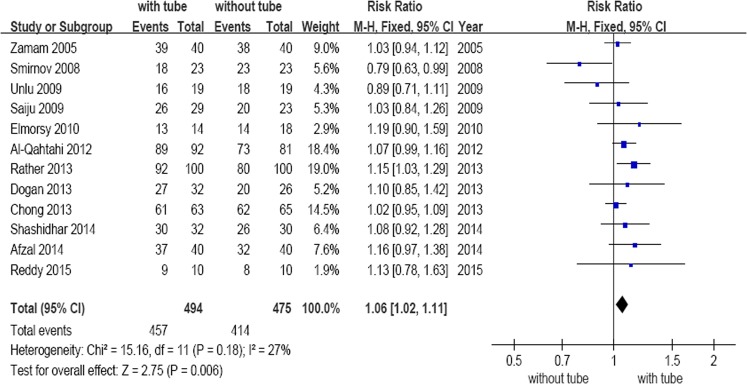
Forest plot: comparison of success rate between DCR with and without silicone intubation.

**Figure 2:**
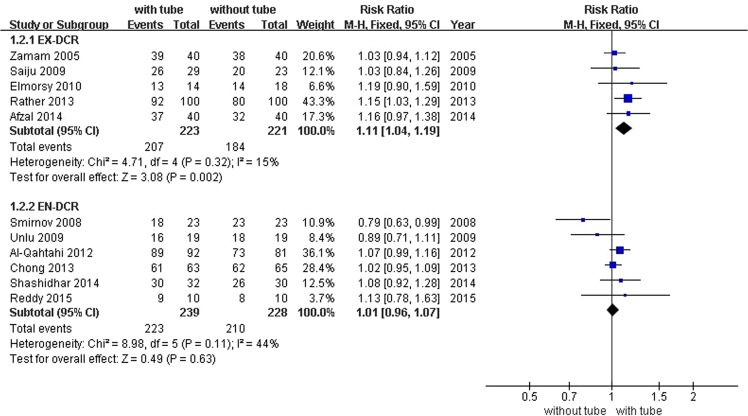
Forest plot: subgroup analysis of the success rate between DCR with and without silicone intubation.

